# Bipartite Medial Cuneiform: A Rare Cause of Chronic Midfoot Pain in a Young Man

**DOI:** 10.7759/cureus.88484

**Published:** 2025-07-22

**Authors:** Alaa Al-Taie, Syeda Shabistan Intekhab, Syed Alam, Renan Ibrahem Adam

**Affiliations:** 1 Radiology, Hamad General Hospital, Doha, QAT; 2 Radiology, Hamad Medical Corporation, Doha, QAT; 3 College of Medicine, Qatar University, Doha, QAT; 4 Musculoskeletal Radiology, Hamad Medical Corporation, Doha, QAT

**Keywords:** anatomical variant, bipartite medial cuneiform, chronic midfoot pain, musculoskeletal radiology, rare anatomical variant

## Abstract

Bipartite medial cuneiform is a rare anatomical variant that results from the failure of fusion between two ossification centers, leading to a bipartition of the medial cuneiform. Although often an incidental finding, it can present with midfoot pain, particularly in the context of acute trauma or chronic overuse. We report the case of a 25-year-old man presenting with chronic, intermittent bilateral midfoot pain. Imaging findings revealed a bilateral bipartite medial cuneiform managed conservatively with orthotics and physiotherapy. This case highlights the importance of identifying this rare anatomical variant and its imaging characteristics to avoid misdiagnosis and guide appropriate management.

## Introduction

The medial cuneiform is part of the distal row of the tarsal bones and is the largest of the three cuneiforms. It articulates proximally with the navicular, laterally with the intermediate cuneiform, and distally with the first and second metatarsal bones. It is part of the medial longitudinal arch and serves as an attachment point for several important tendons such as the tibialis anterior, tibialis posterior, and peroneus longus [1,2]. The Lisfranc joint is a complex articulation involving the medial cuneiform and is an important stabilizing component of the midfoot [3]. 

Bipartite medial cuneiform is a rare anatomical variant of the cuneiform bone seen in approximately 0.1-2.4% of the population [4]. This variant was first described by Barlow in 1942 in an anthropological specimen [5]. Normally, the medial cuneiform develops from a single ossification center beginning around the third year of life. However, in some individuals, two primary ossification centers develop and fail to fuse, resulting in a bipartite medial cuneiform [6]. Although it is mostly an incidental finding, it may occasionally be a source of midfoot pain, particularly in acute trauma or chronic overuse. 

Midfoot pain can result from a wide range of conditions, including common causes such as osteoarthritis, stress fractures, Lisfranc injuries, and accessory bones, as well as rarer entities like symptomatic anatomical variants such as bipartite medial cuneiform [7]. Therefore, it is important to accurately diagnose such patients to avoid unnecessary interventions, especially in rare cases that may be easily overlooked.

We report the rare case of a 25-year-old man with chronic, intermittent bilateral midfoot pain, with imaging findings revealing bilateral bipartite medial cuneiform, successfully managed with conservative treatment.

## Case presentation

A 25-year-old man presented to the orthopedic clinic with a two-year history of intermittent pain in the medial aspect of the left foot. The pain was primarily localized to the left foot; however, he had recently begun to experience mild discomfort in the right foot as well. Symptoms were aggravated by prolonged standing and walking. There was no history of trauma, and the patient had no known comorbidities.

On clinical examination, the patient was noted to have a mild flexible cavovarus deformity of the foot. The subtalar joint demonstrated a full range of pain-free motion. Initial foot radiographs were reported as normal. The patient was started on conservative treatment, including referral to orthotics for custom foot inserts, and an MRI of the left foot was scheduled, along with a follow-up in six months.

**Figure 1:**
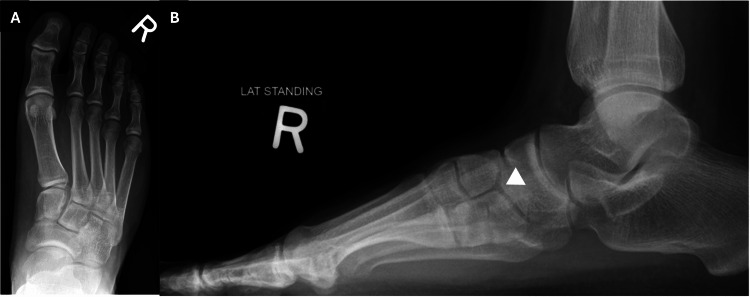
Weight-bearing radiographs of the right foot in anteroposterior view (A) and lateral view (B) showing bipartite medial cuneiform (white arrowhead).

**Figure 2:**
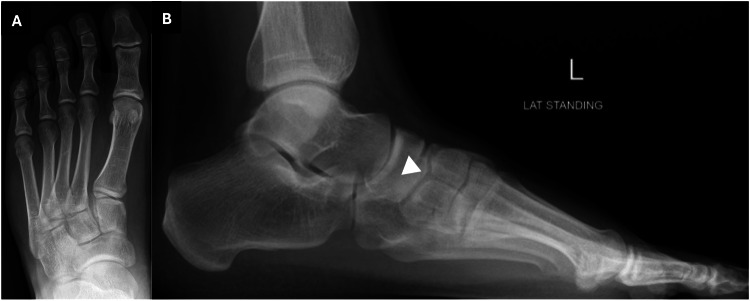
Weight-bearing radiographs of the left foot in anteroposterior view (A) and lateral view (B) showing bipartite medial cuneiform (white arrowhead).

**Figure 3:**
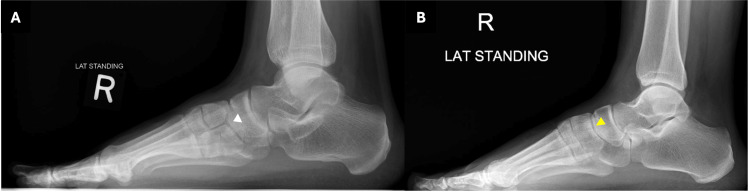
Lateral weight-bearing radiograph with (A) bipartite medial cuneiform (white arrowhead) compared to a (B) normal medial cuneiform in a different patient (yellow arrowhead).

Bilateral foot radiographs were taken as part of the initial assessment. The lateral weight-bearing views demonstrated a lucent line dividing the medial cuneiform into two distinct segments, suggestive of a bipartite medial cuneiform. On the anteroposterior (AP) views, the bipartition was not clearly visualized as the two segments overlapped in this projection (Figure 1 and Figure 2). Figure 3 shows a comparison with a normal radiograph, highlighting the anatomical difference seen in this variant. However, the finding was missed in the initial report, likely due to its rarity. 

**Figure 4:**
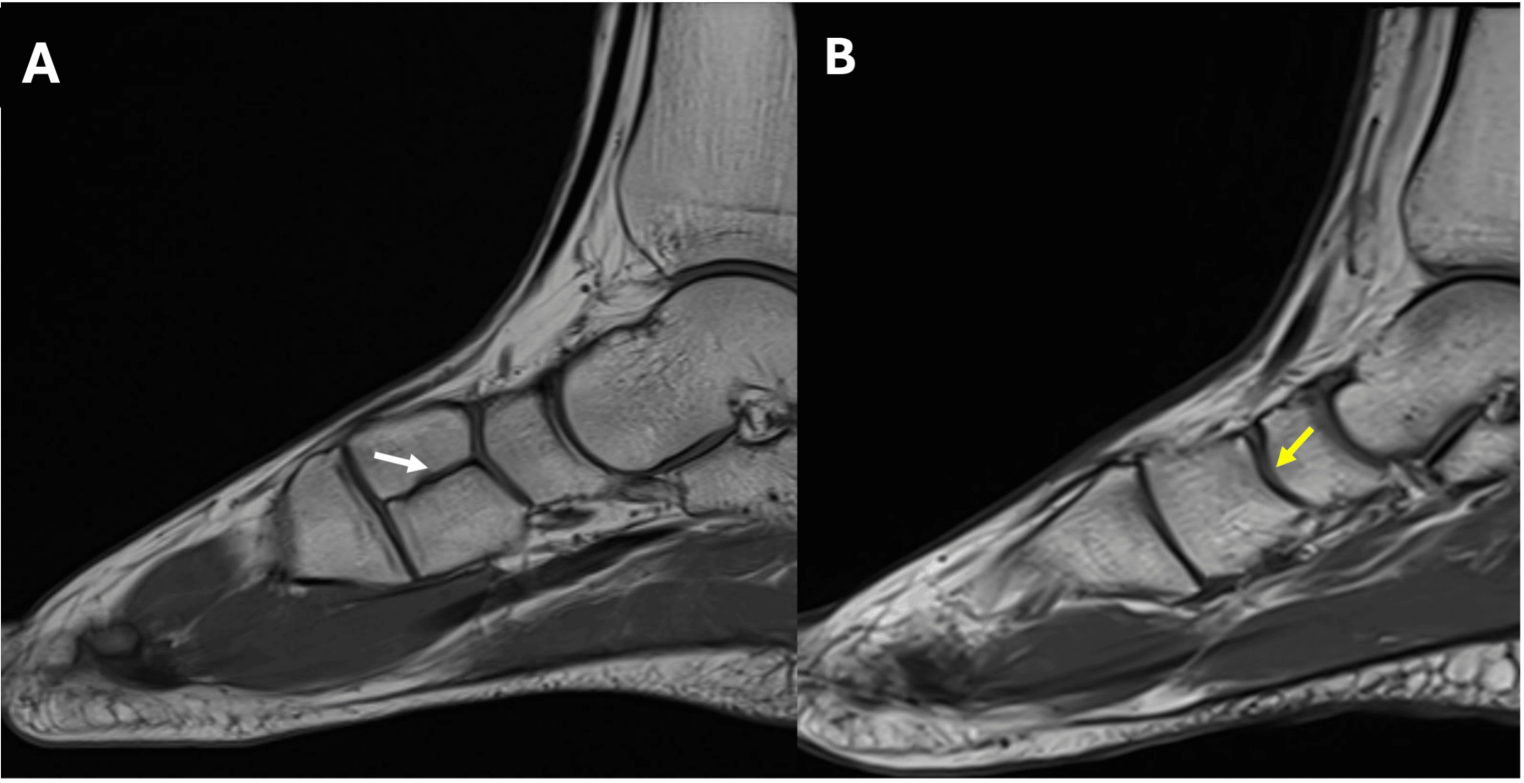
Sagittal T1 MRI: (A) showing dorsal and plantar components of bipartite medial cuneiform (white arrow) articulating with the navicular bone and the base of the first metatarsal bone compared to (B) showing the normal tarsal bone anatomy in a patient without anatomical variation (yellow arrow).

**Figure 5:**
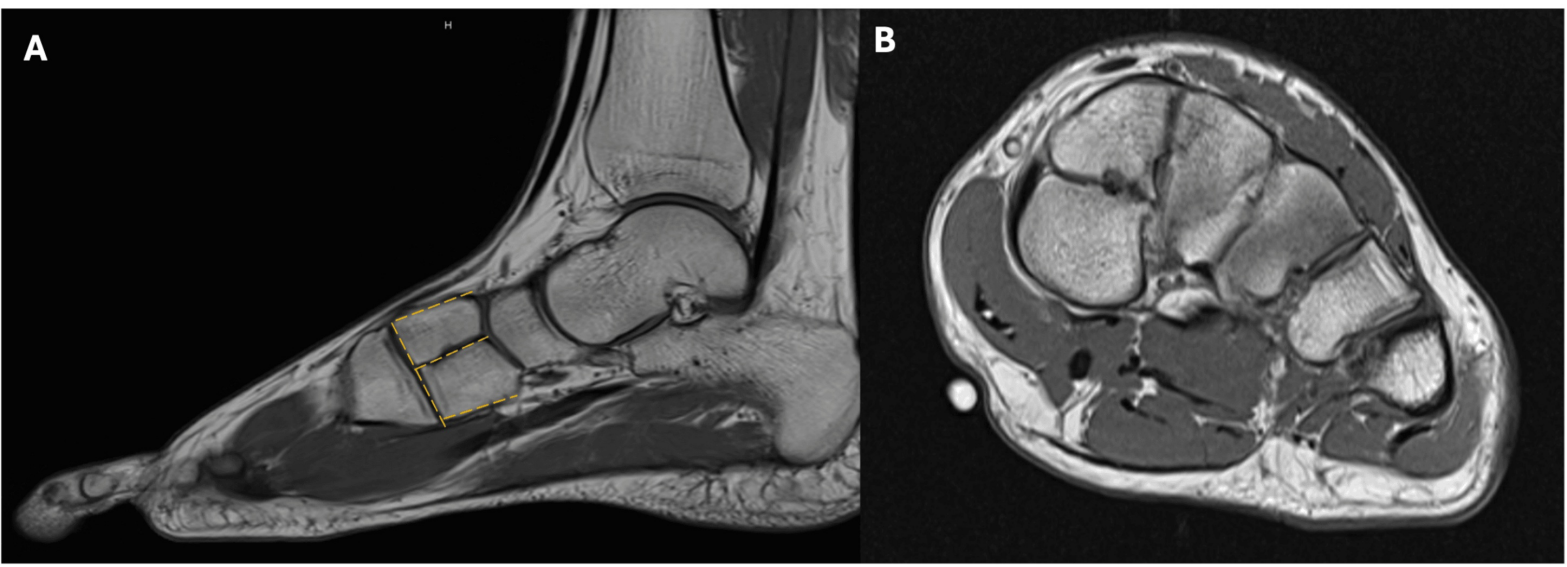
(A) Sagittal T1 MRI of the left foot showing bipartite medial cuneiform with characteristic "E" sign (yellow dashed line). (B) T1 short-axis MRI showing the appearance of bipartite medial cuneiform in short axis.

**Figure 6:**
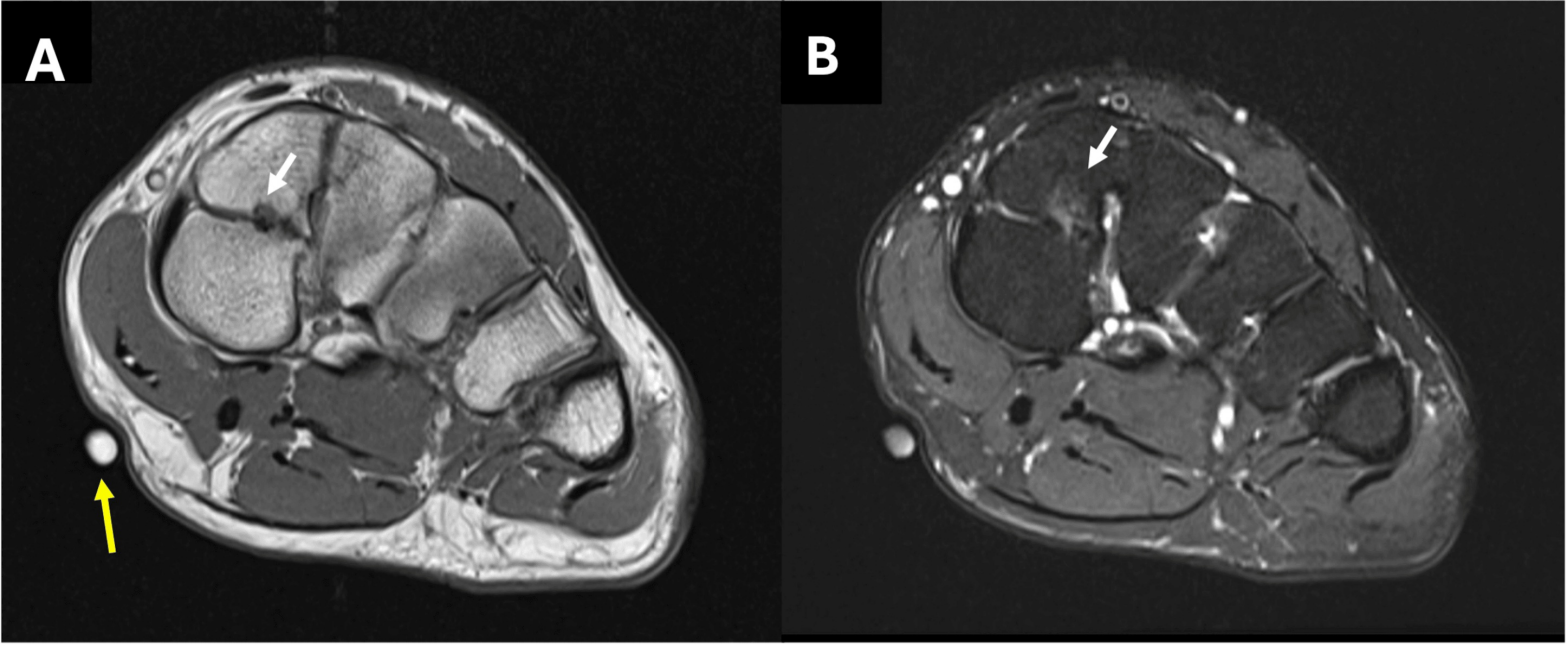
Short-axis T1 MRI (A) and PD FS MRI (B) showing dorsal and plantar components of bipartite medial cuneiform with associated pseudoarthrosis and degenerative changes (white arrows). The marker (yellow arrow) is noted on the medial plantar aspect of the foot directly under the medial cuneiform. PD FS MRI: proton density fat saturation MRI

The patient subsequently underwent MRI of the left foot, which confirmed the diagnosis of bipartite medial cuneiform. The variant anatomy was identified on sagittal and short-axis images, demonstrating distinct dorsal and plantar segments of the medial cuneiform, each articulating with the navicular bone and the base of the first metatarsal bone (Figure 4 and Figure 5). MRI also revealed mild degenerative changes at the pseudoarthrosis between the two segments of the medial cuneiform, likely due to chronic mechanical stress. The localizer indicating the site of pain was located on the plantar aspect of the bipartite medial cuneiform, suggesting that this anatomical variant was the most likely source of the patient's symptoms (Figure 6).

At the six-month follow-up, the patient did not report substantial relief of symptoms and was referred again to orthotics and physiotherapy for further conservative management. At the one-year follow-up, he reported significant improvement, with only occasional pain during long-distance walking. He was able to perform his daily and leisure activities without any restrictions. Therefore, conservative management was deemed sufficient in addressing the patient's symptoms in our case.

## Discussion

Bipartite medial cuneiform is identified when an oblique or horizontal fissure divides the medial cuneiform into two ossicles: a larger plantar segment and a smaller dorsal segment. These two segments may be connected by either a bony or fibrous interface. Aside from the bipartition, no other anatomical differences are typically observed in the insertion of the tendons to the bone. This variant is reported to be bilateral in approximately 60% of cases, possibly indicating a genetic predisposition [8-11]. The bipartition may be either complete or partial, and in bilateral cases, it is more often found to be complete in one foot and partial in the other [8]. In our case, the patient had bilateral bipartite medial cuneiform that was symptomatic on both sides, a finding that has not been reported previously to the best of our knowledge. 

As it is an uncommon anatomical variant, bipartite medial cuneiform may be overlooked on standard radiographs, as the two segments can overlap and obscure the bipartition. A 30-degree oblique view has been reported to improve the visualization of the bipartition on radiographs [12,13]. Being familiar with the imaging features that set this variant apart from normal anatomy can help ensure an accurate diagnosis. In our case, the lateral weight-bearing radiographs demonstrated a lucent line completely dividing the medial cuneiform in both feet, suggestive of a bilateral complete bipartite medial cuneiform. However, a CT or MRI scan may be required to confirm the diagnosis, evaluate the extent of bipartition, and rule out a fracture [10].

On MRI, a well-defined joint space can be observed between the two components of the medial cuneiform and the head of the first metatarsal, giving rise to a characteristic "E"-shaped appearance on sagittal images, which serves as a useful imaging clue for identifying a bipartite medial cuneiform [6,14]. In symptomatic cases such as ours, there may be associated bone marrow edema or surrounding osseous changes, possibly resulting from altered biomechanics [6]. In our patient, the MRI showed bone marrow edema, and the MRI localizer pointed to pain at the plantar aspect of the medial cuneiform, suggesting that the anatomical variant was the most likely cause of his symptoms.

It is essential to differentiate between a bipartite medial cuneiform and a fracture of the medial cuneiform, as both may present with midfoot pain following acute trauma but require different management strategies. One distinguishing feature is the orientation of the dividing plane: in bipartition, the separation is typically horizontal, whereas fractures more commonly exhibit a vertical orientation. A fracture usually has irregular, non-corticated margins, while a bipartite medial cuneiform demonstrates smooth, well-corticated borders. Bone marrow edema is most frequently seen in fractures, but has also been reported in symptomatic cases of bipartite medial cuneiform. Additionally, the combined size of the ossicles in a bipartite cuneiform is generally larger than the individual normal bone, which could be another useful differentiating feature [6,12,13].

Conservative management is often the first-line approach in most cases. In the literature, at least four cases have demonstrated complete resolution of symptoms with conservative treatment alone, including non-steroidal anti-inflammatory drugs, custom foot orthoses, and physiotherapy [10,12,14,15]. Our patient also responded well to conservative management, suggesting that conservative management may be sufficient in most cases. Local steroid injections have been reported to be effective in three prior cases [11,16,17].

A significant proportion of previously symptomatic cases required surgical intervention, particularly in the presence of arthritic changes, persistent bone marrow edema, or failure of conservative management. Surgical options included excision of the smaller fragments, osteosynthesis of the segments, or fusion to adjacent joints [4,11,12,16,18]. 

## Conclusions

Bipartite medial cuneiform is a rare anatomical variant that may present as an incidental finding in asymptomatic individuals or as a cause of midfoot pain, due to either acute trauma or chronic overload. This case highlights the importance of including anatomical variants in the differential diagnosis of persistent midfoot pain and being familiar with characteristic imaging features on radiographs and MRI. Although rare, accurate recognition of this variant is essential to prevent misdiagnosis and avoid unnecessary interventions. Conservative management remains the first-line treatment and is sufficient in most symptomatic cases.
